# Rethinking the objectives of a pan-Canadian immunization information system

**DOI:** 10.17269/s41997-025-01129-y

**Published:** 2025-10-30

**Authors:** Bryan Thomas, Colleen Flood, Kumanan Wilson

**Affiliations:** 1 Faculty of Law, Queen’s University, Kingston, ON Canada; 2 Bruyère Health Research Institute, Ottawa, ON Canada; 3 Department of Medicine, University of Ottawa, Ottawa, ON Canada; 4 Ottawa Hospital Research Institute, Ottawa, ON Canada

**Keywords:** Immunization, Registries, Vaccine safety, Vaccine coverage, Federalism, Constitution, Immunisation, Enregistrements, Innocuité des vaccins, Couverture vaccinale, Fédéralisme, Constitution

## Abstract

Emerging from the pandemic and the largest mass vaccination campaign in history, governments at all levels are once again calling for the development of comprehensive vaccination registries. Yet these calls have persisted for decades at the federal level, and little progress has been made. At the heart of the challenge is the need to clarify the objectives of federal vaccine data collection and how these objectives dovetail with the federal government’s constitutional role and jurisdiction in public health. We suggest that pan-Canadian immunization information collection initially focus on vaccine safety and effectiveness, as these would be most concordant with provincial/territorial aims and would fall under the federal government’s jurisdiction. The federal spending power could be utilized to further support provincial/territorial systems to facilitate pan-Canadian data collection for coverage.

## Résumé

Au sortir de la pandémie et de la plus grande campagne de vaccination de masse de l’histoire, tous les ordres de gouvernement réclament une fois de plus la mise en place de registres de vaccination exhaustifs. Pourtant, bien que ces appels persistent depuis des dizaines d’années au palier fédéral, peu de progrès ont été accomplis. Au cœur de la difficulté, il y a la nécessité de clarifier les objectifs de collecte de données vaccinales fédérales et la concordance de ces objectifs avec le rôle constitutionnel du gouvernement fédéral et sa compétence sur la santé publique. Nous suggérons que la collecte de données d’immunisation pancanadiennes porte initialement sur l’innocuité et l’efficacité des vaccins, car ces deux aspects concorderaient le mieux avec les objectifs provinciaux et territoriaux et relèveraient de la compétence du gouvernement fédéral. Le pouvoir de financement fédéral pourrait servir à soutenir davantage les systèmes provinciaux et territoriaux pour faciliter la collecte de données pancanadiennes afin d’améliorer la couverture.

Emerging from the pandemic and the largest mass vaccination campaign in history—and now facing a resurgence of measles that threatens the Western Hemisphere’s elimination status—governments at all levels are once again calling for the development of comprehensive vaccination registries. Yet these calls have persisted for decades at the federal level, and little progress has been made (Gorfinkel & Lexchin, 2021). At the heart of the challenge is the need to clarify the objectives of federal vaccine data collection and how these objectives dovetail with the federal government’s constitutional jurisdiction in public health. We believe now is the time to rethink the purpose of a federal vaccine data collection system.

Immunization information systems are defined as “A confidential, population-based, computerized information system that attempts to collect vaccination data about all persons within a geographic area.” (Government of Canada, 2023). A vaccine registry, at the provincial/territorial level, may extend beyond this purpose, allowing the use of the data for provision of care at the individual level, for example, identifying and notifying unvaccinated individuals of their status in the event of an outbreak (Personal Health Information Protection Act, 2004). At the federal level, registries have been envisioned primarily to assist with understanding vaccination coverage at a national level as well as within subgroups and exploring geographic and temporal variations. This information can inform federal policies with respect to assisting provincial/territorial governments and communication strategies.

However, this is where the challenge arises. For a federal vaccine registry to succeed, provinces/territories themselves need to have comprehensive data on vaccination, they need to be willing to share this data with the federal government, and there needs to be common standards for immunization data to permit interoperability (Wilson et al., 2017). Unfortunately, there may be a lack of comprehensive vaccination data at the provincial/territorial level. This is particularly true in provinces/territories where physicians are the primary immunizers, as physician electronic medical record systems may not be linked to provincial/territorial immunization data repositories. Even if comprehensive data exists, there is little incentive for a province/territory to share this data with the federal government for coverage assessments, as the data could expose limitations within their jurisdiction and they may perceive that pan-Canadian data does not significantly add to their local coverage assessment efforts. Transfer of provincial/territorial data to the federal level has also been an ongoing challenge in public health beyond immunization (Auditor General of Canada, 2022; Wilson, 2004). With respect to the issue of interprovincial interoperability, progress has been made regarding the standardization of immunization data (Rubens-Augustson et al., 2025).

Furthermore, the extent of federal constitutional jurisdiction over collecting data for vaccine coverage purposes is unclear. During a public health emergency, there can be a clear argument for the federal government to mandate data transfer, although even in these settings the federal government has been reluctant to use its potential constitutional powers—preferring to rely on a collaborative approach with mixed results. During the pandemic, only aggregate COVID-19 vaccine data was shared by the provinces/territories with the federal government, for example.

Absent an emergency, the case for federal powers is more speculative. While the federal government could theoretically invoke various heads of powers to justify its involvement—such as the criminal law power, the quarantine power, the census and statistics power, and the residual “peace, order and good government” (POGG) power—each has serious limitations in this context. Use of the federal *criminal law* power would presumably entail *threatening provincial actors with penalties*—fines or jail time—for refusing to provide vaccination data to the national registry, which seems like a nonstarter given the established norm of cooperative federalism. The Federal Court has interpreted the *quarantine* power as being “conferred specifically for the purpose of preventing or reducing the introduction and spread of communicable diseases from outside the country” (Spencer v. Canada (Health), 2021). So interpreted, it is unlikely that the quarantine power enables routine federal tracking of Canadians’ immunization status where no border crossing is involved. As for the *census* power, there do exist national health registries, for example, the Canadian Cancer Registry, but these are founded on voluntary agreements with the provinces/territories. As with the *criminal law* power, it is far-fetched to imagine the federal government *coercing* provincial/territorial participation in a vaccine registry using the threat of financial penalties contained in the *Statistics Act.* The federal government succeeded in using the *POGG* power to ground its carbon pricing regime. But in allowing this, the Supreme Court of Canada emphasized that the power could only be flexed where provincial inaction had “grave extraprovincial consequences” (Greenhouse Gas Pollution Pricing Act, 2021). It is very doubtful that a national vaccine registry has the requisite urgency. By process of elimination, it therefore seems that the safest path for the federal government, constitutionally, would be to use its spending power to incentivize voluntary provincial/territorial participation in a national vaccine registry. The federal government famously leveraged its spending power, through the *Canada Health Act,* to establish universal health care.

Might the provinces/territories be swayed to participate voluntarily in a national system? There are areas where vaccine data collection is more evidently beneficial to both federal and provincial/territorial orders of government and where there is a clearer mandate for federal government involvement, for example, the post-market surveillance of vaccines. Through the Food and Drugs Act, the federal government has a responsibility to ensure the safety of medicinal products (Food and Drugs Act, 1985). There is also a clear provincial/territorial motivation for sharing vaccine data for safety assessments, as it can result in more rapid ascertainment of potential causal relationships and complement existing vaccine safety systems. For example, the use of pan-Canadian data during the pandemic could have led to more rapid assessments of the risk of myocarditis from mRNA vaccines compared to analyses using only specific provinces’ data (Hawken et al., 2024). Furthermore, a 100% complete data set is not required for vaccine safety—what is more critical is knowing with confidence that a specific individual has been vaccinated and has developed, or not developed, the outcome of interest (Wilson et al., 2012). The United States Vaccine Safety Data Link (VSD), the active surveillance system for vaccine safety in the United States (US), only relies on a fraction of the US population and arguably is not representative as it is based on people enrolled in Health Maintenance Organizations (Centers for Disease Control and Prevention). Determining the association between vaccination and the outcome of interest involves the need to link to other health data sets, something that could be facilitated by the Canadian Institutes of Health Information (CIHI) or other entities that host health administrative data. CIHI has information sharing agreements in place with provinces/territories and already manages multiple health data repositories/registries (Canadian Institute for Health Information, n.d.). The VSD has the advantage of having more detailed information available through medical records, but this is potentially achievable in Canada through linkages with health data record sets such as those housed by systems like GEMINI (Harnessing hospital data to improve healthcare, n.d.). Similarly, post-market surveillance to ensure the ongoing effectiveness of vaccines is of mutual federal and provincial/territorial interest although more robust data is needed for this compared to assessments of vaccine safety, to enable the use of the test-negative design, for example. With existing health administrative data, this would be most useful for establishing protective immunity by demonstrating impact on health services utilization rather than sterilizing immunity, which would require microbiology data to ascertain for laboratory-confirmed outcomes. All of these efforts would require the timely transfer of health administrative data to allow for rapid assessments of safety and effectiveness.

We thus recommend federal officials consider the following (Table 1). Federal or pan-Canadian authorities should work with the provinces/territories to facilitate data transfer initially for the purposes of vaccine safety surveillance. This would ideally be mediated through an intergovernmental agreement where the use of the data can be controlled by the parties sharing the data. A government-adjacent entity such as CIHI may have more success in collecting this data than a purely federal entity given the historical challenges with federal provincial/territorial information-sharing agreements (Wilson et al., 2021). If successful, this approach could then be extended to vaccine effectiveness. In order to move to coverage, investments may need to be made at provincial/territorial levels to ensure comprehensive vaccine data collection. This could be achieved by the use of the federal funding power and the implementation of conditional funding arrangements. The US CDC has used a funding strategy to support the development of state immunization registries which meet federal requirements (Development of community- and state-based immunization registries. CDC response to a report from the National Vaccine Advisory Committee, 2001).

**Table 1:** Pan-Canadian immunization information system

Purpose	Necessary Vaccination Data	Necessary Supporting Data
Phase 1: Vaccine Safety	Administrative or registry data Partial data sufficient. Need to ascertain with confidence an individual received a vaccine and had an outcome of interest	Health administrative data EMR data to permit for more detailed outcomes
Phase 2: Vaccine Effectiveness	Administrative or registry data More comprehensive data collection necessary. Important to ascertain with confidence which individuals received a vaccine and which didn’t.	Health administrative data EMR data to permit for more detailed outcomes Microbiology data to ascertain laboratory-confirmed outcomes if assessing sterilizing immunity
Phase 3: Vaccine Coverage	Registry data with other data sources to fill in gaps Comprehensive data collection needed for accurate assessments	No additional data essential but detailed geographic and demographic data can permit subgroup analyses

The need for pan-Canadian immunization data is particularly urgent given a multitude of new vaccines reaching market or about to reach market, preparations underway for human vaccination against the avian influenza flu vaccine, and the US moving away as a reliable partner in vaccine post-market surveillance and communicable disease surveillance overall (Charlebois & Pawa, 2025; Public Health Agency of Canada, 2024). We believe a new approach needs to be considered with a primary focus on data sharing for the purposes of vaccine safety and effectiveness. None of this is meant to suggest that data collection for vaccine coverage assessments should not be a priority at the provincial or territorial level where the data is critical for both health system planning, public health response, and provision of care at the individual level.

## Data Availability

NA.

## Code availability

NA.
